# Genome-Wide Identification and Analysis of the APETALA2 (AP2) Transcription Factor in *Dendrobium officinale*

**DOI:** 10.3390/ijms22105221

**Published:** 2021-05-14

**Authors:** Danqi Zeng, Jaime A. Teixeira da Silva, Mingze Zhang, Zhenming Yu, Can Si, Conghui Zhao, Guangyi Dai, Chunmei He, Juan Duan

**Affiliations:** 1 Key Laboratory of South China Agricultural Plant Molecular Analysis and Genetic Improvement, Provincial Key Laboratory of Applied Botany, South China Botanical Garden, Chinese Academy of Sciences, Guangzhou 510650, China; zengdanqi20@scbg.ac.cn (D.Z.); zhangmingze@scbg.ac.cn (M.Z.); zhenming311@scbg.ac.cn (Z.Y.); cans2013@163.com (C.S.); zhaoconghui@scbg.ac.cn (C.Z.); 2 College of Life Sciences, University of the Chinese Academy of Sciences, No. 19A Yuquan Road, Shijingshan District, Beijing 100049, China; 3 Independent Researcher, P. O. Box 7, Ikenobe 3011-2, Miki-cho, Kagawa-ken 761-0799, Japan; jaimetex@yahoo.com; 4 Opening Public Laboratory, Chinese Academy of Sciences, Guangzhou 510650, China; daigy@scbg.ac.cn; 5 Center of Economic Botany, Core Botanical Gardens, Chinese Academy of Sciences, Guangzhou 510650, China

**Keywords:** *Dendrobium officinale*, AP2 transcription factor, development, dual-luciferase reporter gene system, gene expression

## Abstract

The APETALA2 (AP2) transcription factors (TFs) play crucial roles in regulating development in plants. However, a comprehensive analysis of the AP2 family members in a valuable Chinese herbal orchid, *Dendrobium officinale*, or in other orchids, is limited. In this study, the 14 DoAP2 TFs that were identified from the *D. officinale* genome and named DoAP2-1 to DoAP2-14 were divided into three clades: euAP2, euANT, and basalANT. The promoters of all *DoAP2* genes contained *cis*-regulatory elements related to plant development and also responsive to plant hormones and stress. qRT-PCR analysis showed the abundant expression of *DoAP2-2*, *DoAP2-5*, *DoAP2-7*, *DoAP2-8* and *DoAP2-12* genes in protocorm-like bodies (PLBs), while *DoAP2-3*, *DoAP2-4*, *DoAP2-6*, *DoAP2-9*, *DoAP2-10* and *DoAP2-11* expression was strong in plantlets. In addition, the expression of some *DoAP2* genes was down-regulated during flower development. These results suggest that *DoAP2* genes may play roles in plant regeneration and flower development in *D. officinale*. Four *DoAP2* genes (*DoAP2-1* from euAP2, *DoAP2*-*2* from euANT, and *DoAP2-6* and *DoAP2-11* from basal ANT) were selected for further analyses. The transcriptional activation of DoAP2-1, DoAP2-2, DoAP2-6 and DoAP2-11 proteins, which were localized in the nucleus of *Arabidopsis thaliana* mesophyll protoplasts, was further analyzed by a dual-luciferase reporter gene system in *Nicotiana benthamiana* leaves. Our data showed that pBD-DoAP2-1, pBD-DoAP2-2, pBD-DoAP2-6 and pBD-DoAP2-11 significantly repressed the expression of the LUC reporter compared with the negative control (pBD), suggesting that these DoAP2 proteins may act as transcriptional repressors in the nucleus of plant cells. Our findings on *AP2* genes in *D. officinale* shed light on the function of *AP2* genes in this orchid and other plant species.

## 1. Introduction

As relatively static organisms, biochemical and genetic mechanisms in plants tend to be sophisticated, including delicate networks involved in regulatory mechanisms that allow plants to adapt to varying environments and resist biotic and abiotic stresses. Transcription factors (TFs) are proteins that can bind to DNA in a sequence-specific manner to regulate transcription. The regulation of gene transcription by TFs is an extremely complicated process [1], and is vital to plant growth and environmental responses. APETALA2 (AP2) belongs to the APETALA2/Ethylene Response Factor (AP2/ERF) superfamily [2,3], which participates in the regulation of various biological processes in plants, such as growth and development (flower development, somatic embryogenesis, meristem and leaf growth, etc.), hormones and stress responses [4,5,6,7,8,9,10,11,12,13].

Historically, the AP2/ERF superfamily has been divided into four separate families, namely the ERF, AP2, RAV and Soloist families [3,10]. The AP2 protein is exclusive to plants and contains two AP2 domains that have also been found in ancient plants such as gymnosperms, mosses, and *Chlamydomonas*, indicating that AP2 and EREBP (ethylene responsive element binding protein) families differentiated before Chlorophyta and Streptophyta lineages differentiated [14]. There are also similarities, including structures and conserved motifs, among the four families, suggesting that they have similar properties. For example, three *A. thaliana* AP2 genes (*AtAP2-6*, *AtAP2-7* and *AtAP2-11*) are involved in the regulation of seed development [15].

TFs recognize target DNA sequences with different DBDs, thereby controlling the expression of target gene promoters at the transcriptional level [16]. The AP2 domain not only plays a key role in transcriptional regulation [7], but also serves as the basis for family classification. In particular, the AP2 family, which contains two AP2 domains and a small number of proteins with a single AP2 domain [2,3,10,17], was further subdivided into the euAP2 (which is characterized by the miR172 binding motif) and AINTEGUMENTA (ANT) (which is characterized by signature amino acid insertions in the AP2 domain) clades [18,19] based on the amino acid sequences and nuclear localization of the two AP2 domains. Among them, the ANT clade was further divided into the basalANT and euANT clades. euANT proteins are defined by a long pre-domain region and four conserved motifs, which is the main difference between basalANT and euANT clades [19,20]. In total, based on differences in gene structure, plant AP2 proteins are divided into three subfamilies: euAP2, euANT and basalANT in the model plant *Arabidopsis thaliana* [20].

Early studies found that in *A. thaliana*, *AP2* was a homeotic gene with a profound effect on floral organs that could determine the identity and fate of floral organs [21,22,23,24]. In rapeseed (*Brassica napus*), the *AP2*-like gene *BABY BOOM* regulates somatic embryogenesis [5]. In addition, the ANT clade is involved in the development of various plant organs, such as vegetative organs [25] and ovule development [26] in *A. thaliana*, fruit development in apple [27], berry size in grapevine [28], and many other examples. In addition, the rice *AP2*-like gene *SNB*, which belongs to the euAP2 clade, regulates the development of grains, such as seed shattering [29], *Eriobotrya japonica* EjAP2-1 interacts with EjMYB to induce fruit lignification [30], while the maize (*Zea mays*) *AP2* genes *ids1* and *sid1* regulate the initiation of corn flower meristems to determine the fate of meristem cells [31]. These studies demonstrate that the AP2 family is involved in the regulation of processes associated with plant development, such as flower development, embryonic development, meristem and leaf growth, among others.

Even though the AP2 family in plants has been extensively studied, AP2 family members in the medicinal (herbal) orchid, *Dendrobium officinale*, i.e., DoAP2s, have not been analyzed. The identification and analysis of DoAP2 family members will not only provide clues for revealing the function of AP2s in *D. officinale*, but also provide a theoretical basis for studying the functional conservation of AP2/ERF TFs, such as identifying new conserved protein domains and motifs, and enriching the functions of TF families in plants. In this study, 14 *DoAP2* genes were identified from *D. officinale*. They were systematically analyzed, a phylogenetic tree was constructed, protein interactions were predicted, and promoter *cis*-acting elements were analyzed, including their subcellular localization and using a dual-luciferase reporter assay. Moreover, using quantitative real-time polymerase chain reaction (qRT-PCR), their expression patterns were analyzed at different developmental stages, including flower development, and in response to different stress treatments, to lay a theoretical foundation for further analyzing the functions of DoAP2s in *D. officinale*.

## 2. Results

### 2.1. Identification and Analysis of AP2 Gene Family in D. officinale

**Figure 1:**
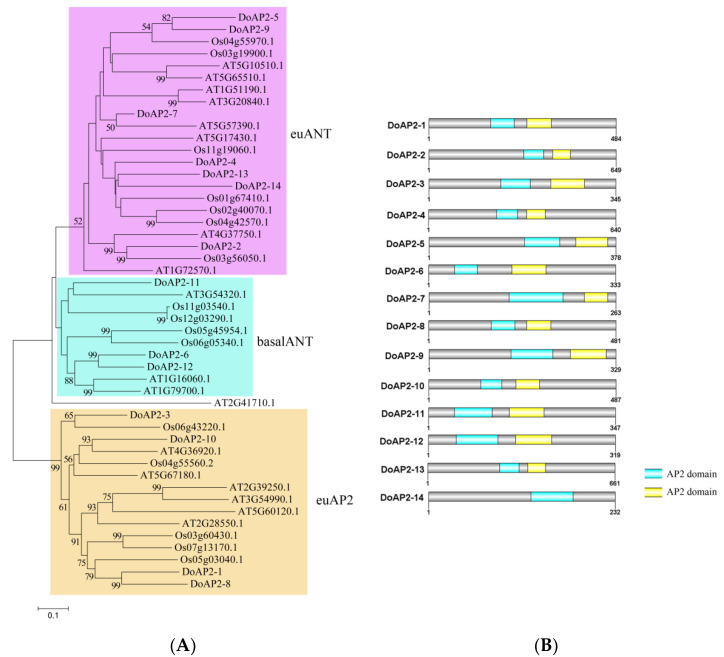
Phylogenetic analysis and conserved domain of the AP2 proteins from *D*. *officinale*, *O. sativa* and *A*. *thaliana*. (**A**) Phylogenetic tree of DoAP2 proteins. A total of 14 AP2 proteins from *D*. *officinale*, 16 from *O. sativa* and 18 from *A*. *thaliana* were aligned using ClustalX to generate a FASTA alignment file. The phylogenetic tree was constructed using the MEGA 7.0 program and the neighbor-joining (NJ) method with 1000 bootstrap replications based on the alignment file; (**B**) Conserved domain of DoAP2 proteins. The location and size of AP2 domains are shown by different colors.

A total of 14 genes (*DoAP2-1* to *DoAP2-14*) annotated as AP2 TFs were identified from the *D*. *officinale* genome. All 14 AP2 proteins from *D*. *officinale*, 16 AP2 proteins from *Oryza sativa*, and 18 AP2 proteins from *A*. *thaliana* were used to perform a phylogenetic analysis. The DoAP2 proteins were classified into three clades: euAP2, euANT, and basalANT (Figure 1A). The euANT clade contained the most (seven) AP2 proteins (DoAP2-2, DoAP2-4, DoAP2-5, DoAP2-7, DoAP2-9, DoAP2-13 and DoAP2-14), followed by the euAP2 clade with four members (DoAP2-1, DoAP2-3, DoAP2-8 and DoAP2-10), while only three AP2 proteins (DoAP2-6, DoAP2-11 and DoAP2-12) were found in the basalANT clade. The AP2 domain is responsible for DNA binding and protein complex formation of AP2 proteins [32]. Two AP2 domains were present in 13 of the 14 DoAP2 proteins, whereas DoAP2-14 only contained one AP2 domain (Figure 1B and Figure S1).

### 2.2. Prediction of Protein–Protein Interaction Network of AP2 Proteins

**Figure 2:**
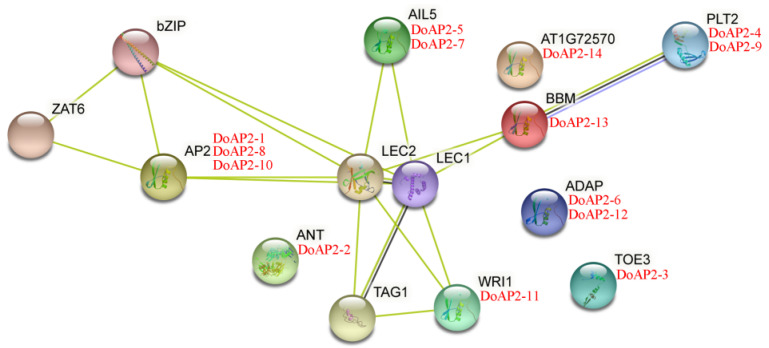
Protein–protein interaction network analysis of DoAP2 proteins using STRING 11. Cyan line represents data from curated databases, green lines indicate gene neighborhoods, and black lines indicate co-expression. Red text = DoAP2s; black text = the proteins in *A. thaliana*.

Protein–protein interactions play a role in transcriptional activation/repression and serve crucial functions in cellular regulation and biological processes in plants. Hence, we analyzed the protein–protein interaction network of the 14 DoAP2 proteins by STRING 11 and found that they were analogous to the interactions displayed by the corresponding *A. thaliana* orthologous proteins (Figure 2). These results show that several members of the DoAP2 family may have a certain connection to LEC1 and LEC2 of the LEC protein family, which is involved in embryonic development [33]. Interestingly, DoAP2-2, DoAP2-3, DoAP2-6, DoAP2-12, and DoAP2-14 were not linked to, nor did they interact with, any other DoAP2s based on the protein–protein interaction network of DoAP2s. Some DoAP2 proteins interacted with other DoAP2 proteins, such as DoAP2-4 and DoAP2-13 while others interacted with TFs involved in plant growth and development, such as DoAP2-1 and b-ZIP. These interactions suggest that DoAP2s play a broad role in plant growth and development.

### 2.3. Analysis of Cis-Regulatory Elements in the Promoters of DoAP2 Genes

**Figure 3:**
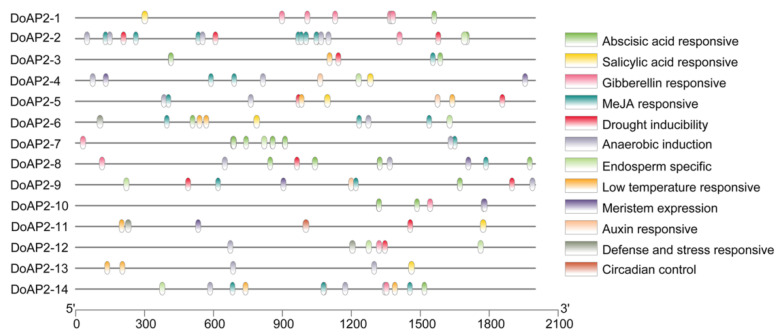
Prediction of *cis*-responsive elements in the 2-kbp upstream region of the initiation codon of 14 *DoAP2* genes. Different colored boxes indicated different *cis*-responsive elements. MeJA, methyl jasmonate.

*AP2* genes that are involved in plant growth and development are regulated by different factors. In order to investigate the *cis*-regulatory elements in the promoters of *DoAP2* genes, we isolated the 2000-bp upstream section according to the *D*. *officinale* genome and analyzed the *cis*-regulatory elements using the PlantCARE web site. The *cis*-regulatory elements of the promoters of *DoAP2* genes were related to growth and development (meristem expression and specific to the endosperm), plant hormones (auxin, abscisic acid, methyl jasmonate (MeJA), gibberellin, and salicylic acid) and stress (drought inducibility, low temperature responsiveness, anaerobic induction, and defense and stress responsiveness). As depicted in Figure 3, more than half of the *DoAP2* genes harbored a total of seven meristem expression-responsive elements and eight endosperm specific-responsive elements, indicating that *DoAP2* genes may play a vital role in meristem growth and embryonic development of *D. officinale*. In addition, an abundance of elements responsive to plant hormones was present in the promoters of all *DoAP2* genes, demonstrating the response of these genes to these hormones. Interestingly, MeJA-responsive elements formed the largest group of elements among the promoters of *DoAP2* genes, indicating that *DoAP2* genes are MeJA-responsive genes (Figure 3). As a vital cellular regulator, MeJA plays a crucial role in mediating various developmental processes and defense responses against biotic and abiotic stresses [34]. Furthermore, except for *DoAP2-10*, the remaining 13 genes contained a total of 45 abiotic stress-responsive elements, not only suggesting that the expression of 13 *DoAP2* genes was associated with these abiotic stresses, but also that they played a role in various stress regulatory networks. Collectively, these results indicate that *AP2* family members participate in embryonic development, meristem growth and environmental stress regulation during the growth and development of *D. officinale*.

### 2.4. Expression Analysis of DoAP2 Genes at Different Developmental Stages

**Figure 4:**
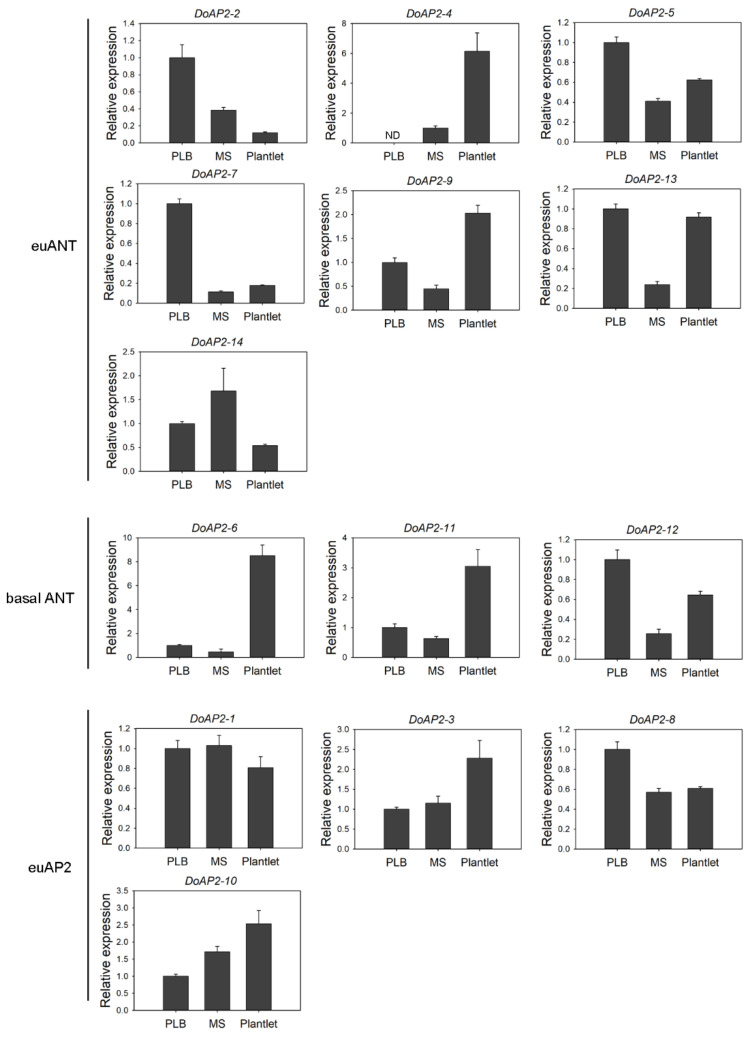
Expression analysis of *DoAP2* genes during plant regeneration from PLBs by qRT-PCR. PLB, protocorm-like bodies; MS, multiple shoots. Each data bar represents the mean ± standard deviation (SD) of three biological replicates (*n* = 3). ND, not detected.

AP2 TFs are regarded as factors that are primarily responsible for the regulation of developmental programs [10]. Protocorm-like bodies (PLBs), which form during the in vitro culture of orchid plants, can proliferate or develop into a complete plant. We analyzed the changes in expression of *DoAP2* genes during the development of PLBs to plantlets (PLBs, multiple shoots and plantlet). All *DoAP2* genes were detected at all three developmental stages, except for *DoAP2*-*4*, which showed the highest expression in PLBs and the lowest expression in plantlets (Figure 4). The decrease in expression from PLBs to plantlets suggests that *DoAP2*-*4* may play a role in PLB development. In addition, *DoAP2*-*7* and *DoAP2*-*8* were strongly detected in PLBs (Figure 4). *DoAP2*-*3*, *DoAP2*-*4*, *DoAP2*-*6*, *DoAP2*-*9*, *DoAP2*-*10*, and *DoAP2*-*11*, but especially *DoAP*-*6*, were abundant in plantlets (Figure 4). The expression of *DoAP2*-*1* was not different among the three developmental stages. The *DoAP2* genes displayed different expression patterns, even within the same clade. For example, in the euANT clade, *DoAP2*-*2* and *DoAP2*-*7* were highly expressed in PLBs while *DoAP2*-*4* and *DoAP2*-*9* were highly expressed in plantlets (Figure 4).

**Figure 5:**
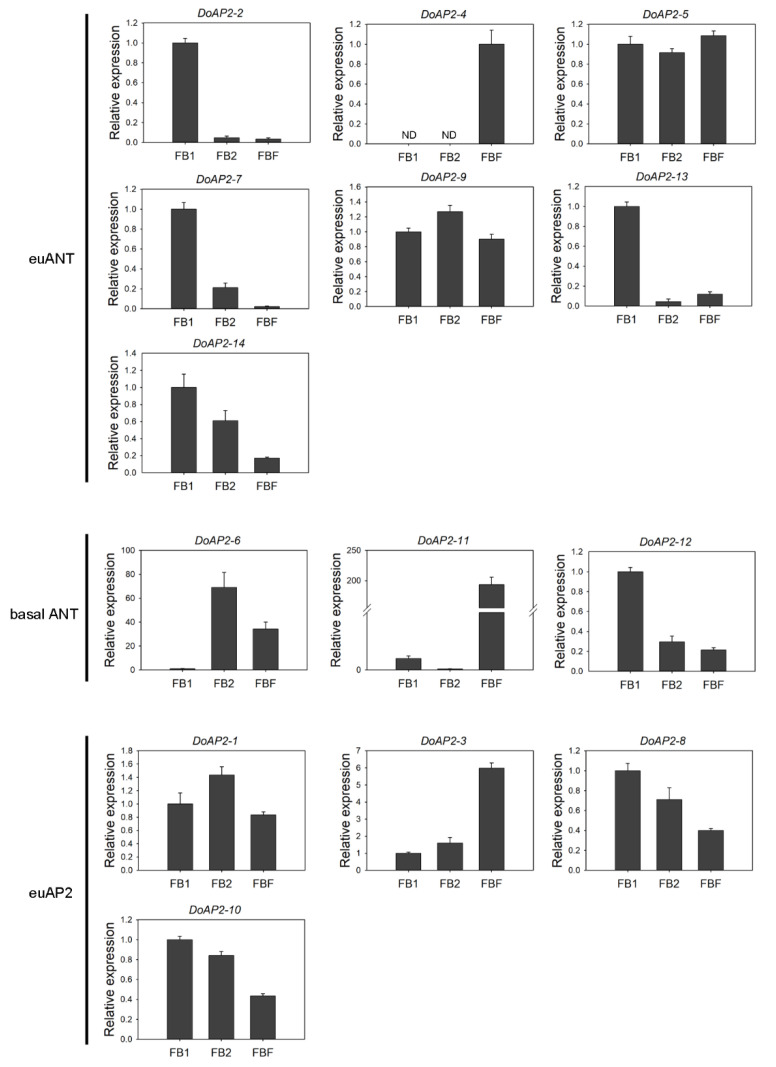
Expression analysis of *DoAP2* genes during flower development by qRT-PCR. FB1, small flower buds (about 5 mm long); FB2, medium flower buds (about 10 mm long); FBF, fully bloomed flowers. Each data bar represents the mean ± standard deviation (SD) of three biological replicates (*n* = 3). ND, not detected.

Flowers are important functional organs of plants. AP2 is involved in flower development [23]. For example, two genes, *ANT* and *ANT*-*LIKE6*, regulate *A. thaliana* floral growth and patterning [35]. We detected the expression of *DoAP2* genes during three stages of *D*. *officinale* flower development, in small flower buds (FB1), medium flower buds (FB2), and fully bloomed flowers (FBF) (Figure 5). As shown in Figure 5, *DoAP2*-*8* and *DoAP2*-*10* exhibited a similar expression pattern, showing relatively high expression levels in FB1, decreasing as the flower developed further. The expression of *DoAP2*-*3*, which was in the same clade as *DoAP2*-*8* and *DoAP2*-*10*, was up-regulated during flower development, and most expressed in FBF. A similar pattern was found in *DoAP2*-*2*, but a different expression pattern in *DoAP2*-*4* and *DoAP2*-*5*, all from the euANT clade (Figure 5). Four out of seven euANT genes were abundant during FB1 (Figure 5). In particular, *DoAP2*-*11* was specifically expressed in FBF, the expression of *DoAP2*-*11* in FBF was about 193.56- and 2225.64-fold higher than in FB1 and FB2 while its expression level was much higher than that of other *DoAP2* genes. These findings show the specificity of expression of different *DoAP2* genes in floral development. Moreover, seven *DoAP2* genes (*DoAP2*-*2*, *DoAP2*-*7*, *DoAP2*-*8*, *DoAP2*-*10*, *DoAP2*-*12*, *DoAP2*-*13*, and *DoAP2*-*14*) had the highest expression in FB1 compared to FB2 and FBF, suggesting that they might play an essential role in the pre-flowering developmental state where differentiation is not yet complete. These results suggest that *DoAP2* family members play a role in the development of *D. officinale* flowers.

The unique flower shape of orchids gives them high ornamental value [36]. We then analyzed the expression of *DoAP2* genes in different floral tissues (sepal, petal, lip, and column) at the FBF stage (Figure S2). The expression of *DoAP2*-*4* and *DoAP2*-*13* was not detected in any of the four tissues of FBFs, and *DoAP2*-*2*, *DoAP2*-*7*, and *DoAP2*-*14* were expressed only in the column and might be closely related to flower morphogenesis. Interestingly, the aforementioned genes that were specifically expressed, or not expressed, belong to the euANT clade. Remarkably, the remaining nine *DoAP2* genes were expressed in different tissues of FBFs. Among them, *DoAP2*-*1*, *DoAP2*-*3*, and *DoAP2*-*11* were more highly expressed in petals, and the expression trends of *DoAP2*-*1* and *DoAP2*-*3* of the same clade were consistent; *DoAP2*-*6* was the most highly expressed in the column, the expression of the column was about 12.88-, 5.88- and 7.72-fold higher than in the sepal, petal and lip, respectively; the expression of *DoAP2*-*5*, *DoAP2*-*9*, *DoAP2*-*10*, and *DoAP2*-*12* were all abundant and the variations were small among the four tissues of FBF. Based on these findings, *DoAP2* genes had unique expression patterns, indicating that they probably played diverse roles in different *D. officinale* FBF tissues.

### 2.5. Expression Analysis of DoAP2 Genes in Response to Abiotic Stresses

**Figure 6:**
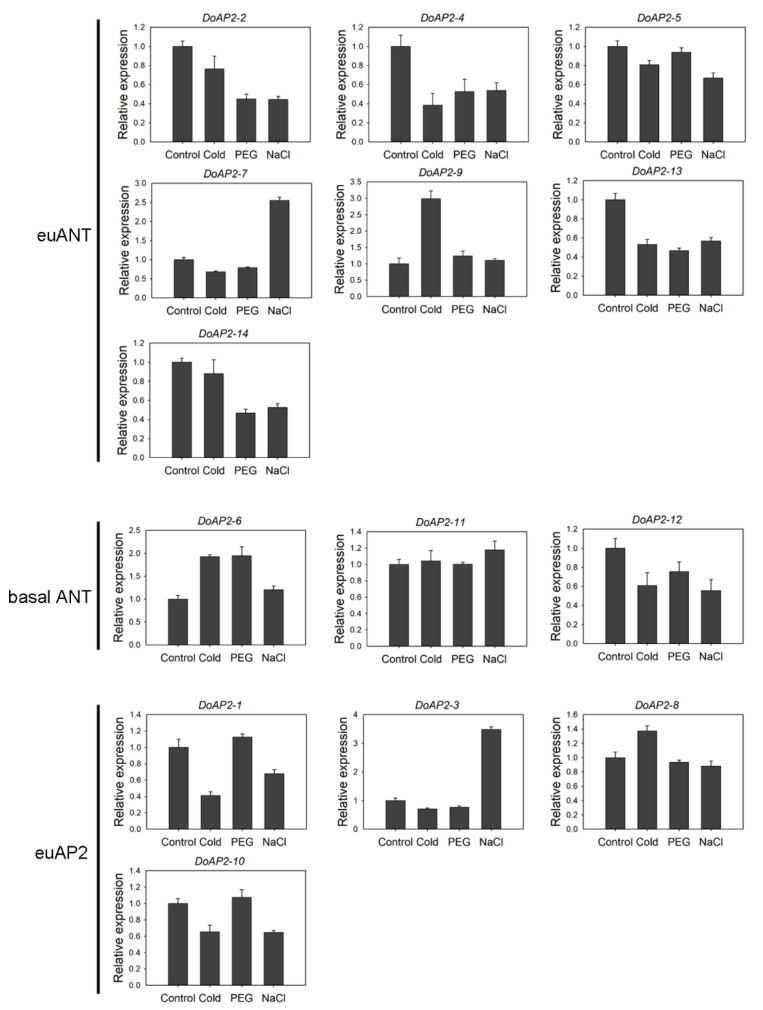
Expression analysis of *DoAP2* genes in response to abiotic stresses (cold, PEG and NaCl) by qRT-PCR. PEG treatment, 15% polyethylene glycol (PEG)-6000; NaCl treatment, 250 mM NaCl; cold treatment, 4 °C. Each data bar represents the mean ± standard deviation (SD) of three biological replicates (*n* = 3).

*AP2* responds to abiotic stresses [37]. To further investigate the changes in expression of *DoAP2* genes to different abiotic stress treatments (cold, PEG and NaCl), we examined the expression levels of 14 *DoAP2* genes under abiotic stress using qRT-PCR, while samples from untreated plantlets served as the control (Figure 6). According to their expression profiles, the expression of *DoAP2-2*, *DoAP2-4*, *DoAP2-5*, *DoAP2-12*, *DoAP2-13*, and *DoAP2-14* were reduced to varying degrees. In addition, the differences in expression of *DoAP2-11* in different treatments were slight, indicating that the above treatments had little effect on *DoAP2-11*. The expression of *DoAP2-3* and *DoAP2-7* in the NaCl treatment was about 3.49- and 2.56-fold higher than in the control, demonstrating that they played a role in the mechanism of response to stress in the face of adversity, especially high salt stress. The expression of *DoAP2-9* in the cold treatment was more abundant than in the other three treatments. Compared to the control, the expression of at least one of the remaining *DoAP2* genes was up-regulated in response to these abiotic stress treatments. In particular, the expression of *DoAP2-6* was 1.93-, 1.95- and 1.21-fold higher than the control in the cold, PEG and NaCl treatments, respectively, implying its potential importance in the adaptation of this orchid to adverse growth conditions experiencing abiotic stresses.

### 2.6. Subcellular Localization of Selected DoAP2 Proteins

**Figure 7:**
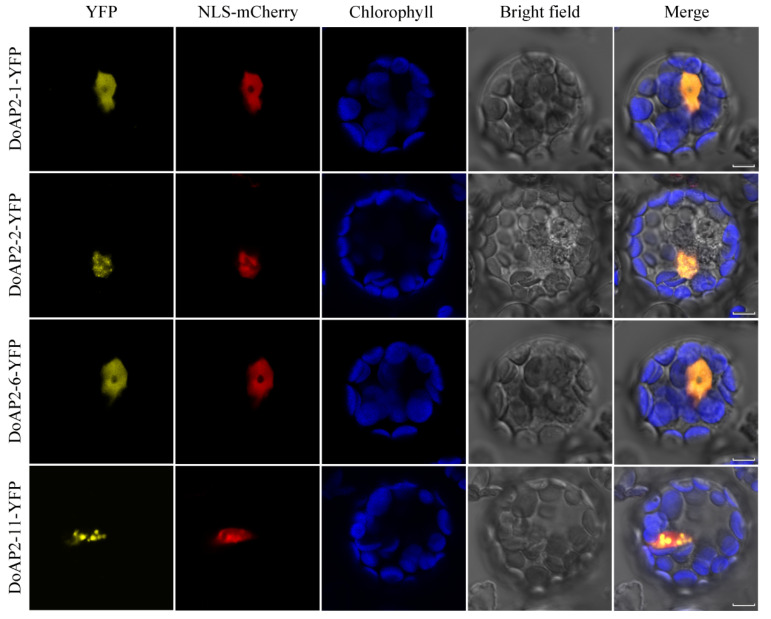
Subcellular localization of four DoAP2 proteins (DoAP2-1, DoAP2-2, DoAP2-6 and DoAP2-11) in *A. thaliana* protoplasts. Bars = 5 μm.

To explore the localization of DoAP2 proteins, *A. thaliana* protoplasts were PEG-mediated transformed with a transient expression vector containing YFP. According to the phylogenetic tree (Figure 1), AP2 proteins of *D. officinale* and *A. thaliana* were classified into three clades. We selected four representative genes, namely DoAP2-2 of the euANT clade, DoAP2-6 and DoAP2-11 of the basalANT clade, and DoAP2-1 of the euAP2 clade, for further analysis. Subcellular localization was observed using a Zeiss LSM 510 Meta confocal microscope. Yellow fluorescence signals of the positive control (empty YFP vector) were detected in the cytoplasm and plasma membrane (Figure S3). As expected, the yellow fluorescent signals of four YFP-DoAP2-fused proteins were localized in the nucleus (Figure 7), conforming to their transcriptional regulatory function in the nucleus.

### 2.7. Four DoAP2 Proteins Displayed Transcriptional Repression in Tobacco Leaves

**Figure 8:**
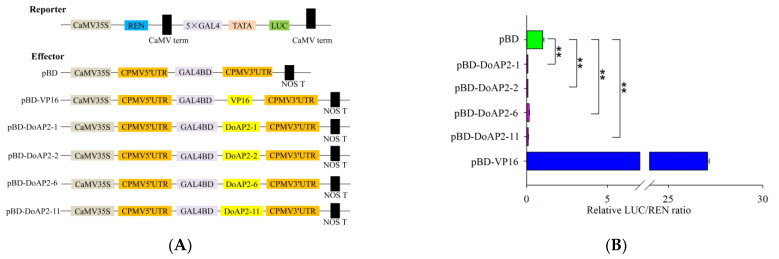
Transcriptional activity assay of *DoAP2* genes in tobacco leaves. (**A**) Schematic presentation of the reporter and effector vectors; (**B**) Transcriptional repression ability of DoAP2 proteins in tobacco leaves. Double asterisks (**) indicate significant differences in two-treatment comparisons (*p* < 0.01) using Dunnett’s test, compared with the negative control (pBD). Each value represents the means of six biological replicates.

AP2 family members are considered to be TFs, and a defining feature of a TF is its transactivation activity [38]. Hence, we investigated the transcriptional activity of four *DoAP2* genes (*DoAP2-1*, *DoAP2-2*, *DoAP2-6* and *DoAP2-11*) using a dual-luciferase reporter gene system in tobacco (*Nicotiana benthamiana*) leaves. These representative genes are the same as those that were used for subcellular localization. The constructed vectors are shown in Figure 8A. We used vectors containing the CaMV35S-driven pBD and fusion protein vectors pBD-VP16, pBD-DoAP2s (DoAP2-1, DoAP2-2, DoAP2-6 and DoAP2-11) as the effector, and the CaMV35S-driven LUC and TATA cassette-driven REN as reporters. pBD-EMPTY and pBD-VP16 were used as negative and positive controls, respectively. In this system, the reporter vector was generated by fusing the *firefly luciferase* (*LUC*) gene after five GAL4 binding sites, and a *renilla luciferase* (*REN*) gene driven by a *CaMV35S* promoter in the reporter vector was used as the internal control (Figure 8A). The ORF of the four *DoAP2* genes was cloned into the site of the pBD vector, which is after the GAL4 binding domain (Figure 8A). The ratio of the two luciferases (LUC and REN) was detected using a dual fluorescent reporter gene system assay (Figure 8B). The LUC/REN ratio of the positive control pBD-VP16 was 27-fold higher than the negative control pBD, while the LUC/REN ratio of the four pBD-DoAP2 proteins was significantly lower than that of pBD (Figure 8B). These results show that *DoAP2-1*, *DoAP2-2*, *DoAP2-6*, and *DoAP2-11* genes had transcriptional repression activity in tobacco plants, i.e., they are transcriptional repressors.

## 3. Discussion

### 3.1. Bioinformatics Analysis of DoAP2 TFs

In this study, we identified 14 *AP2* genes in the *D. officinale* genome. The AP2 family is a small TF family with fewer members in plants than the ERF TF family. For example, 18 *AP2* genes were found in model plant Arabidopsis [3], 26 in *Indica* rice [39], and 62 in wheat [40]. The AP2 proteins from plants are divided into three subfamilies: euAP2, euANT and basalANT [10,14,20,41]. In this study, DoAP2 proteins were classified into euAP2, euANT and basalANT subfamilies, similar to other plants. All AP2 proteins contain two AP2 domains except for DoAP2-14, which has a single AP2 domain (Figure 1B and Figure S1). This is consistent with a prior finding that AP2 family members contain one or two AP2 domains [10].

A study of protein interactions can enrich the characteristics of TFs, such as localization, transcription activity, target specificity and function. Based on the STRING 11 tool, all protein–protein interaction networks of DoAP2 proteins were predicted. Interestingly, some DoAP2 proteins may interact with TFs involved in plant growth and development such as b-ZIP. Additionally, there are many reports on the interaction of proteins with members of the AP2/ERF superfamily. For example, rice OsERF3 interacted with WOX11 to regulate rice crown root development [42], AP2 TF HaDREB2 in sunflower (*Helianthus annuus* L.) interacted with another TF HaHSFA9 to regulate zygotic embryogenesis [43], and AtERF5 in *A. thaliana* interacted with AtERF6, AtERF8, SCL13 and other proteins to exert a wide range of regulatory effects, such as defense against phytopathogenic fungi [44,45]. The results of interaction protein prediction in this paper (Figure 2) provide a notion about the possible binding nature of DoAP2 proteins, although further verification is needed with yeast two hybrid, co-immunoprecipitation and bimolecular fluorescence complementation assays to determine the DoAP2 protein interaction network and its protein interaction under different conditions.

Increasing lines of evidence have shown that the AP2/ERF superfamily is mainly involved in development and abiotic stress responses [5,7,8,13,46,47]. In the present study, many growth and development, hormone and stress *cis*-acting elements were detected in the promoter regions of *DoAP2* genes (Figure 3). In addition, *cis*-acting elements regulate the expression of stress-inducible genes, leading them to be referred to as molecular switches that regulate various biological processes [48]. The promoters of *DoAP2* genes contain endosperm-specific *cis*-acting elements with expression in the meristem in response to growth and developmental processes, and that respond to multiple stress signals, which might regulate various biological processes. This is consistent with the basic functions of AP2 TFs [37,49]. Different DoAP2 TFs play a variety of roles that may be related to their specific and/or differential binding to different *cis*-acting elements or other proteins, suggesting their involvement in different regulatory processes [50,51,52].

### 3.2. The AP2 TF Play Important Roles in Plant Regeneration and Flower Development

An increasing number of studies provide evidence for the involvement of *AP2* genes in plant regeneration and flower development.

In plant regeneration, AP2/ERF superfamily TFs promoted callus induction and proliferation, shoot differentiation, root differentiation and differentiation of somatic cells [53]. Several examples are provided next. Overexpression of the AP2 gene *ZmBBM2* promoted callus induction and proliferation in maize [54]. In cacao (*Theobroma cacao*), overexpressed of the *TcBBM* gene induced embryo formation; moreover, the *TcBBM* gene can be used as an embryogenesis biomarker in cacao [11]. *HbAP2-3* and *HbAP2-7* genes are marker genes of somatic embryogenesis during callus proliferation in rubber tree (*Hevea brasiliensis*) [55]. An AP2/EREBP-type transcription activator NtCEF1 regulated gene expression in tobacco callus [56]. In rapeseed and *A. thaliana*, BBM, which shares similarities with the AP2/ERF superfamily of TFs, led to the differentiation of somatic cells, inducing embryonic development [5]. An AP2/ERF TF WOUND INDUCED DEDIFFERENTIATION1 (WIND1) promoted shoot regeneration in *A. thaliana* [57]. In *A. thaliana*, AP2/ERF TFs play an important role in root regeneration [58]. The AP2/ERF gene *GmRAV1* regulated the regeneration of roots and adventitious buds in soybean [59]. *D. officinale* PLBs are considered to be somatic embryos that can proliferate and also differentiate into complete plants [60]. The induction, proliferation and regeneration of PLBs is an advantageous method for the large-scale production of *D. officinale* [61]. In addition, AP2 TFs specify the identity of floral organs and regulate the expression of genes related to flower development [20,21,22,23,24,62,63]. For example, *D. officinale* flowers contain three petalized sepals, two petals, one lip and one column [64], the unique floral patterning gives this orchid its ornamental value, it provides advantages to pollination, and promotes normal plant development [65]. In this study, there were five *DoAP2* genes (*DoAP2-2*, *DoAP2-5*, *DoAP2-7*, *DoAP2-8* and *DoAP2-12*) that showed abundant expression in PLBs from among the three development stages of *D. officinale*. In particular, it is important to emphasize that we found that *DoAP2-2* and *DoAP2-7* were specifically expressed in PLBs while *DoAP2-2* was down-regulated during the development of *D. officinale*. Seven *DoAP2* genes (*DoAP2-2*, *DoAP2-7*, *DoAP2-8*, *DoAP2-10*, *DoAP2-12*, *DoAP2-13* and *DoAP2-14*) were strongly expressed in the early flower buds, and were down-regulated as flowers developed (Figure 5), suggesting that *DoAP2* genes play a role in *D*. *officinale* flower development. Among them, the expression levels of *DoAP2-2* and *DoAP2-7* were highest in PLBs and FB1, and both strongly expressed in the column (Figure S2), indicating that *DoAP2-2* and *DoAP2-7* are highly specific genes that may play an important role in immature tissues of *D. officinale*. These findings imply that *DoAP2* and *DoAP2-7* genes are involved in regulating the maintenance of immature tissues and flower development, supporting the view that AP2 TFs have important functions in regulating plant growth and development. The expression levels of *DoAP2-6* and *DoAP2-11* of the same clade basal ANT were similar at different stages (Figure 4), and their expression continued to increase during development, peaking in plantlets and FBF. However, they also displayed some differences. Among the expression levels of different tissues in FBF, *DoAP2-6* showed abundant expression in the column, while *DoAP2-11* expression was abundant in sepals and petals. In *D. officinale*, *DoAP2* genes have diverse roles in flowers, similar to the expression pattern of the *NsAP2* gene in floral organs of water lily (*Nymphaea* sp. cv. ‘Yellow Prince’) [49]. Despite these similarities, flower development is a very complicated process, and *AP2* genes may be directly or indirectly involved in a certain regulatory role. Therefore, it is necessary to conduct more in-depth and detailed research on these genes.

### 3.3. DoAP2 Genes May Play a Role in Abiotic Stress Response

The expression of *DoAP2-6* increased to varying degrees, especially in response to cold stress and PEG treatment (Figure 6). The expression of *DoAP2-11* also increased, but its amplitude was much lower than that of *DoAP2-6* (Figure 6). This shows that the basal clade members *DoAP2-6* and *DoAP2-11* may have important regulatory effects under adverse abiotic stresses, allowing for a response to salt and drought stress during the growth and development of *D. officinale* plants. *DoAP2-6* and *DoAP2-11* contained a large number of stress-related *cis*-acting elements related to drought and low temperature (Figure 3), which may be closely related to their increased expression levels under different stress treatments (Figure 6), indicating that they are involved in the regulation of adverse abiotic stresses. Collectively, the above results suggest that genes with similar *cis*-acting elements among genes of the same clade may perform similar functions [66].

### 3.4. The DoAP2 Proteins Are Localized in the Nucleus and Display Transcription Activity

Nuclear localization is a key regulatory mechanism of TFs [67]. Our subcellular localization analysis of DoAP2 proteins indicated that, like many other AP2 and ERF TFs such as PsAP2 [68], OsDREBL [69] and GsERF71 [70], DoAP2-1, DoAP2-2, DoAP2-6 and DoAP2-11 were localized in the nucleus (Figure 7). These findings demonstrate that DoAP2 proteins have the basic characteristics of TFs, performing functions in the nucleus. However, since we selected representative genes of each clade from among the 14 DoAP2 TFs, this does not mean that all DoAP2 proteins have the above characteristics, and the related characteristics of the remaining proteins still needs additional research.

Based on their functions, TFs can be divided into either activators or repressors. Repressors play an important role in the regulation of gene expression by inhibiting the expression of certain genes by combining with DNA elements, transcription activators or promoter sequences [71], enabling plants to save energy under normal (non-adverse) conditions [72]. The expression of a repressor is also closely related to growth and development, and overexpression of repressors can lead to abnormal plant development [73,74]. However, compared with activators, there is less research on suppressors, especially in non-model plants. Previous studies showed that *AP2* genes can negatively regulate the expression of certain genes to achieve corresponding functions, and this has been well studied in *A. thaliana*. For example, early studies found that *A. thaliana* AP2 is a negative regulator of the AGAMOUS gene, both of which were involved in flower development, and the regulatory mechanism of their interaction established the expression pattern of floral homologous genes in *A. thaliana* to some extent [75]. An *A. thaliana AP2* gene (*At4g36920*) negatively regulated the *REPLUMLESS* (*RPL*) gene that controls fruit dehiscence to achieve the function of controlling fruit development [62]. The *AP2* gene negatively regulates the size and number of embryonic cells, thus achieving the function of affecting seed mass and seed yield in *A. thaliana* [15,76]. Moreover, the AP2-like TF mutant of rice showed enlarged grains and increased grain weight [29], and *AP2* genes also negatively regulated the formation of the abscission layer [77], thereby affecting the development of rice grains. In this study, we further tested the transcription activity of DoAP2 TFs using the dual-luciferase assay. As shown in Figure 8, *DoAP2* genes had a strong repressive effect. This is likely to be related to the AP2/EREBP domain, which belongs to the DBD of a plant transcription repressor [32]. Such a repressor needs to bind to DNA to repress transcription [78]. In short, studies of repressors have important biological significance, and they can not only enrich our understanding of the negative regulatory role of plants in response to external environmental stresses, but also provide new theoretical guidance for genetic improvement of plant resistance to stresses in adverse growth conditions. Therefore, further in-depth studies on DoAP2 TFs will be of great significance for breeding and trait improvement.

## 4. Materials and Methods

### 4.1. Plant Material and Growth Conditions

The *D. officinale* plants used in this study were grown and maintained in the South China Botanical Garden, Chinese Academy of Sciences, Guangzhou, China. The expression patterns of *DoAP2* genes were performed on different *D. officinale* tissues (see below). The sampling method is also described below. PLBs, multiple shoots (MS, i.e., without roots) and plantlets (about 5 cm high) of *D. officinale* were grown on half-strength Murashige and Skoog (1/2MS) [79] medium supplemented with 20 g/L sucrose, 6 g/L agar and 1 g/L activated carbon (pH 5.4) in a growth chamber. We collected *D. officinale* material treated with PEG, NaCl and cold stress. Among them, concentrations were selected based on a relevant previous study [80]. First, *D. officinale* plants under normal growth conditions were cultured on 1/2MS with 0.1% activated carbon, 2% sucrose, and 0.6% agar medium (pH 5.4). *D. officinale* plants were then separately exposed to one of several abiotic stresses (on the basis of the above growth conditions): 15% polyethylene glycol (PEG)-6000 (Sigma-Aldrich, Shanghai, China; the PEG treatment), 250 mM NaCl (Guangzhou Chemical Reagent Factory, Guangzhou, China; the NaCl treatment) and 4 °C (the cold treatment). The culture conditions in controlled-climate chambers were: 26 ± 1 °C, 86.86 μmol·m^-2^·s^-1^, a 12-h photoperiod, and about 60% relative humidity. In addition, MS and plantlets were derived from PLBs, as PLBs can grow into MS and plantlets after culture in the above medium. Each treatment was conducted as three replications and five *D. officinale* PLBs, MS and plantlets were used for each treatment. All samples were instantaneously frozen in liquid nitrogen for 15 min then stored at −80 °C for later use.

### 4.2. Identification of DoAP2 Genes from the D. officinale Genome

From the NCBI (https://ftp.ncbi.nlm.nih.gov, accessed on 27 September 2020) genome database, we selected *D. officinale* and downloaded the *D. officinale* genome file. The AP2 protein sequences from *O. sativa* and *A. thaliana* were obtained from Plant Transcription Factor Database (http://planttfdb.gao-lab.org/index.php, accessed on 9 October 2020). Genes were identified by a hidden Markov model (HMM) search based on the AP2 domain using the Pfam protein domain database (http://pfam.xfam.org/, accessed on 10 October 2020). The HMM file was assessed by the HMMER3 software package under default parameters (http://hmmer.janelia.org/, accessed on 10 October 2020). Subsequently, DoAP2 proteins were verified via a local HMM-based search program (E-value ≤ 1 × 10^−10^). Furthermore, the identified sequences were confirmed to be AP2 proteins by annotation as an AP2 protein, either in the Uniprot database (https://www.uniprot.org/, accessed on 11 October 2020) or in the NCBI database. Retrieved AP2 protein sequences were compared with the *A. thaliana* AP2 protein sequences and a phylogenetic tree was constructed using the MEGA version 7 program [81]. Lastly, the remaining 14 proteins were considered to be *D. officinale* DoAP2 proteins.

### 4.3. Bioinformatics Analysis of DoAP2 Proteins

DNAMAN version 8.0 software (Lynnon Biosoft, Foster City, CA, USA) was used to generate multiple sequence alignments of full-length amino acid sequences of the DoAP2 proteins. In addition, we also used Clustal X 2.0 [82] to perform multiple alignments of DoAP2 proteins to further verify the results of sequence alignment. For the phylogenetic analysis, based on the alignment of AP2 proteins, we used MEGA version 7 [81] to perform phylogenetic and molecular evolutionary analyses of AP2 proteins. Initially, the amino acid sequences of the AP2 proteins from *D. officinale* and *A. thaliana* (*FASTA* format) were arrayed with Clustal X 2.0 [82], and the UniProt BLAST online website (http://www.uniprot.org/blast/, accessed on 11 October 2020) was used to calculate sequence identity based on the neighbor-joining method [83] with 1000 bootstrap replicates. Thus, a phylogenetic tree of *D. officinale* and *A. thaliana* AP2s was constructed. *D. officinale* AP2 proteins were categorized by their phylogenetic relationships with the corresponding *A. thaliana* AP2 proteins. Additionally, we used NCBI’s conserved domain database (CDD) [84] to identify the conserved domains of DoAP2 proteins, and to calculate the conserved domain start sites and lengths. Finally, DOG2.0 software (http://dog.biocuckoo.org/, accessed on 22 October 2020) was used to map the distribution of conserved domains.

We obtained the promoter sequences of *DoAP2* genes (Table S1) from the *D. officinale* whole genome sequencing files. Subsequently, the upstream 2000 bp sequence relative to the translation initiation codon (ATG) of the promoter of each *DoAP2* gene was selected as the promoter region, and the PlantCare online software (http://bioinformatics.psb.ugent.be/webtools/plantcare/html/, accessed on 19 November 2020) was used to predict the *cis*-acting elements in the promoters of *DoAP2* genes. Finally, the prediction map of *DoAP2* genes’ promoters were drawn by TBtools [85].

Based on the association model of *A. thaliana*, the STRING 11 tool (https://string-db.org, accessed on 2 December 2020) [86] was used to predict the protein–protein interaction network between DoAP2 proteins and other proteins.

### 4.4. RNA Extraction, cDNA Synthesis and qRT-PCR

The RNA extraction kit, RNAout2.0 reagent (Tiandz Inc., Beijing, China) was used to extract total RNA from the aforementioned *D. officinale* materials according to the operation manual. We used RNase-free DNase I (Takara Bio Inc., Kyoto, Japan) to purify RNA. After the extracted RNA was digested with DNase, 2 μL was applied to agarose gel electrophoresis, and the integrity of the RNA was detected by the Clinx GenoSens gel documentation system (Clinx Science Instruments, Shanghai, China). Total RNA was reverse transcribed using the GoScript™ Reverse Transcription System (Promega, Madison, WI, USA) in accordance with the manufacturer’s protocol, and 4 µg of purified total RNA was used for reverse transcription. The reaction system was 20 µL to synthesize first strand cDNA. The obtained cDNA was diluted in ddH_2_O to 1:50 and applied as a template for qRT-PCR analysis. The LightCycler 480 system (Roche, Basel, Switzerland) that uses the Aptamer™ qPCR SYBR^®^ Green Master Mix (Tianjin Novogene Bioinformatics Technology Co. Ltd., Tianjin, China) was used to perform qRT-PCR. Reaction conditions were: 95 °C for 5 min, and 40 subsequent cycles of 95 °C for 10 s and 60 °C for 1 min. *D*. *officinale ACTIN* (NCBI accession number: JX294908) was used as the internal reference gene [87] to standardize cDNA concentration. Relative gene expression was calculated using the 2^−ΔΔC^^T^ method [88]. Supplementary Table S2 lists the specific primer sequences for the *DoAP2* genes. Three independent biological replicates were performed for each sample.

### 4.5. Subcellular Localization Analysis

The transient gene expression system that uses *A. thaliana* mesophyll protoplasts is an advantageous tool, and is often used for subcellular localization. First, we inserted the entire coding sequence of the four *DoAP2* genes (*DoAP2-1*, *DoAP2-2*, *DoAP2-6* and *DoAP2-11*) without stop codons into the *Eco*RI site of the pSAT6-EYFP-N1 vector [89]. In addition, this study used an additional method [90] to isolate protoplasts from *A. thaliana* leaves at the 4-weeks-old stage. Since TFs are generally located in the nucleus, for further verification, the recombinant protein was combined with the NLS location marker to transform *A. thaliana* mesophyll protoplasts using a PEG-mediated method [90]. After incubation for 12–18 h in the dark, a Leica TCS SP8 STED 3× microscope (Wetzlar, Hesse, Germany) was used to excite the YFP fluorescence signal at 514 nm to observe the yellow fluorescence signal of *A. thaliana* mesophyll protoplasts. The primers used to construct the four YFP- DoAP2 fused proteins are listed in Supplementary Table S3.

### 4.6. Dual-Luciferase Reporter (DLR) Assay

The DLR assay, which was used to investigate the transcriptional activation of TFs, was performed according to a previous report [91]. Briefly, the coding sequences of *DoAP2* (*DoAP2-1*, *DoAP2-2*, *DoAP2-6* and *DoAP2-11*) genes without the stop codon were inserted into the constructed pBD vector driven by the 35S promoter as effector, and the double-reporter vector as reporter, which includes a GAL4-LUC and an internal control REN driven by the 35S promoter. The effector and reporter were genetically transformed into tobacco (about 5–6 weeks, young non-flowering plants) leaves using *Agrobacterium tumefaciens* strain GV3101 (Weidi, Shanghai, China) and tobacco plants were cultured in the dark for 3 days at 25 °C. Finally, according to the manufacturer’s instructions, the activities ratio of the two luciferases (LUC and REN) was carried out using the DLR assay (Promega Corp., Madison, WI, USA) and measured using a GloMax 20/20 luminometer (Promega Corp.). The results were calculated as the ratio of LUC to REN. Six independent biological replicates were performed. At least six transient assay measurements were performed for each assay. The primers used to construct the four pBD-DoAP2 fusion constructs are listed in Supplementary Table S4.

### 4.7. Statistical Analysis

In figures, data have been plotted as means ± standard deviation (SD). Analysis of variance (ANOVA) followed by the Dunnet test was used to determine significant differences at *p* < 0.05 and *p* < 0.01. Analyses were conducted with SPSS v. 22.0 software (IBM) for Windows (IBM Corp., Armonk, NY, USA).

## 5. Conclusions

We identified 14 DoAP2 TFs from a precious Chinese herbal medicinal orchid, *D. officinale*. We analyzed the expression of *DoAP2* genes in different tissues of *D. officinale* and provided evidence for the specific expression and important regulatory roles at different developmental stages. Promoter analysis of *DoAP2* genes showed that they contained a large number of *cis*-acting elements related to development and abiotic stress, supporting the diversity of their regulatory functions. Results of the protein interaction prediction helped to find putative functions for the DoAP2 TFs, providing a basis for further analysis and verification. Moreover, qRT-PCR analysis showed that *DoAP2* genes are involved in regulating many biological processes such as floral development, embryonic development and adversity to stress, thus play a variety of roles in *D. officinale*. Importantly, use of CLSM to observe the subcellular localization of DoAP2-1, DoAP2-2, DoAP2-6, and DoAP2-11 allowed for the verification that all were localized in the nucleus. Furthermore, the DLR assay demonstrated that DoAP2-1, DoAP2-2, DoAP2-6, and DoAP2-11 proteins displayed strong transcription inhibitory activity in *Nicotiana benthamiana*, indicating that they are transcriptional repressors that inhibit expression. Overall, our study shows not only that DoAP2 TFs have transcriptional inhibitory activity, but also that they were mainly involved in regulating different growth and development stages of *D. officinale*, especially flower development. Our results have relevance to genetically modified resistant breeding since *DoAP2* genes were expressed in PLBs, flowers, and plantlets, and were involved in biological processes such as stress response.

## Data Availability

Not applicable.

## Supplementary Materials

The following are available online at https://www.mdpi.com/article/10.3390/ijms22105221/s1, Figure S1. Multiple sequence alignment of all DoAP2 proteins using ClustalX 2.1, Figure S2. Expression analysis of DoAP2 genes in different tissues of FBF by qRT-PCR, Figure S3. Subcellular localization of positive control (empty YFP vector) in *A. thaliana* protoplasts, Table S1. Promoter sequences of *DoAP2* genes, Table S2. Primers used for qRT-PCR, Table S3. Primers used for subcellular localization analysis, Table S4. Primers used for the dual-luciferase reporter gene system.

## Abbreviations

1/2MS	half-strength Murashige and Skoog (1962) medium
AD	transcriptional activation domain
AP2	APETALA2
AP2/ERF	APETALA2/Ethylene Response Factor
BD	DNA-binding domain
CDD	conserved domain database
Co	column
DBD	DNA binding domain
EREB	ethylene response element binding factor
FB1	small flower bud
FB2	medium flower bud
FBF	fully bloomed flower
HMM	Hidden Markov mode
Li	lip
*LUC*	*firefly luciferase*
MEGA	Molecular Evolutionary Genetics Analysis
MS	multiple shoots
PEG	polyethylene glycol
PLB	protocorm-like body
Pe	petal
qRT-PCR	quantificational real-time polymerase chain reaction
*REN*	*renilla luciferase*
Se	sepal
TF	transcription factor
YFP	yellow fluorescent protein
