# Supplementary material for: Genome-Wide Identification and Analysis of the APETALA2 (AP2) Transcription Factor in Dendrobium officinale
Source: Int J Mol Sci. 2021 May 14;22(10):5221. doi: 10.3390/ijms22105221 (PMC8156592; doi:10.3390/ijms22105221)
Supplement: Supplementary file 1 [file ijms-22-05221-s001.zip › ijms-1197157-for conversion-sup.pdf]

International Journal of *Molecular Sciences*

MDPI

Article

Supplementary Materials

Genome-Wide Identification and Analysis of the APETALA2 (AP2) Transcription Factor in *Dendrobium officinale*

Danqi Zeng [1,2], Jaime A. Teixeira da Silva [3], Mingze Zhang [1,2], Zhenming Yu [1], Can Si [1], Conghui Zhao [1,2], Guangyi Dai [4], Chunmei He [1,*] and Juan Duan [1,5,*]

[1] Key Laboratory of South China Agricultural Plant Molecular Analysis and Genetic Improvement, Provincial Key Laboratory of Applied Botany, South China Botanical Garden, Chinese Academy of Sciences, Guangzhou 510650, China; zengdanqi20@scbg.ac.cn (D.Z.); zhangmingze@scbg.ac.cn (M.Z.); zhenming311@scbg.ac.cn (Z.Y.); cans2013@163.com (C.S.); zhaoconghui@scbg.ac.cn (C.Z.)
[2] College of Life Sciences, University of the Chinese Academy of Sciences, No. 19A Yuquan Road, Shijingshan District, Beijing 100049, China
[3] Independent Researcher, P. O. Box 7, Ikenobe 3011-2, Miki-cho, Kagawa-ken 761-0799, Japan; jaimetex@yahoo.com
[4] Opening Public Laboratory, Chinese Academy of Sciences, Guangzhou 510650, China; daigy@scbg.ac.cn
[5] Center of Economic Botany, Core Botanical Gardens, Chinese Academy of Sciences, Guangzhou 510650, China
* Correspondence: hechunmei2012@scbg.ac.cn (C.H.); duanj@scib.ac.cn (J.D.); Tel.: +86-20-37252993 (J.D.); Fax: +86-20-37252978 (J.D.)

Citation: Zeng, D.; Teixeira da Silva, J.A.; Zhang, M.; Yu, Z.; Si, C.; Zhao, C.; Dai, G.; He, C.; Duan, J. Genome-Wide Identification and Analysis of the APETALA2 (AP2) Transcription Factor in Dendrobium officinale. *Int. J. Mol. Sci.* **2021**, *22*, 5221. https://doi.org/10.3390/ijms22105221

Academic Editor: Lam-Son Phan Tran

Received: 12 April 2021
Accepted: 11 May 2021
Published: date

Publisher's Note: MDPI stays neutral with regard to jurisdictional claims in published maps and institutional affiliations.

Copyright: © 2021 by the authors. Submitted for possible open access publication under the terms and conditions of the Creative Commons Attribution (CC BY) license (https://creativecommons.org/licenses/by/4.0/).

Supplementary Figure legends

Figure S1. Multiple sequence alignment of all DoAP2 proteins using ClustalX 2.1.

Figure S2. Expression analysis of DoAP2 genes in different tissues of FBF by qRT-PCR.

Figure S3. Subcellular localization of positive control (empty YFP vector) in *A. thaliana* protoplasts.

Supplementary Table legends

Table S1. Promoter sequences of *DoAP2* genes.

Note: Because the promoter sequences in "**Table S1**" are too long to display in Word format, they are represented in **Table S1** as a separate Excel table ("Supplementary Material-Table S1 Promoter sequence of DoAP2 genes.xlsx").

Table S2. Primers used for qRT-PCR.

Table S3. Primers used for subcellular localization analysis.

Table S4. Primers used for the dual-luciferase reporter gene system.

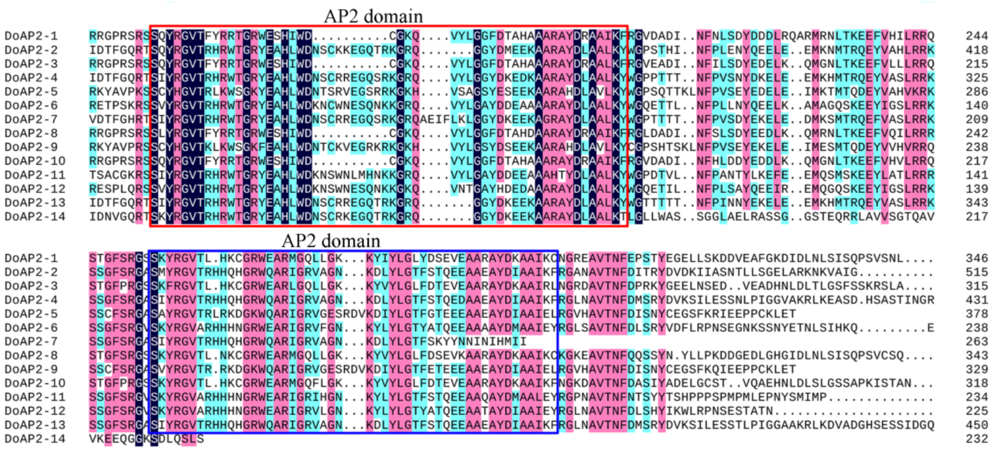

Figure S1. Multiple sequence alignment of all DoAP2 proteins using ClustalX 2.1.

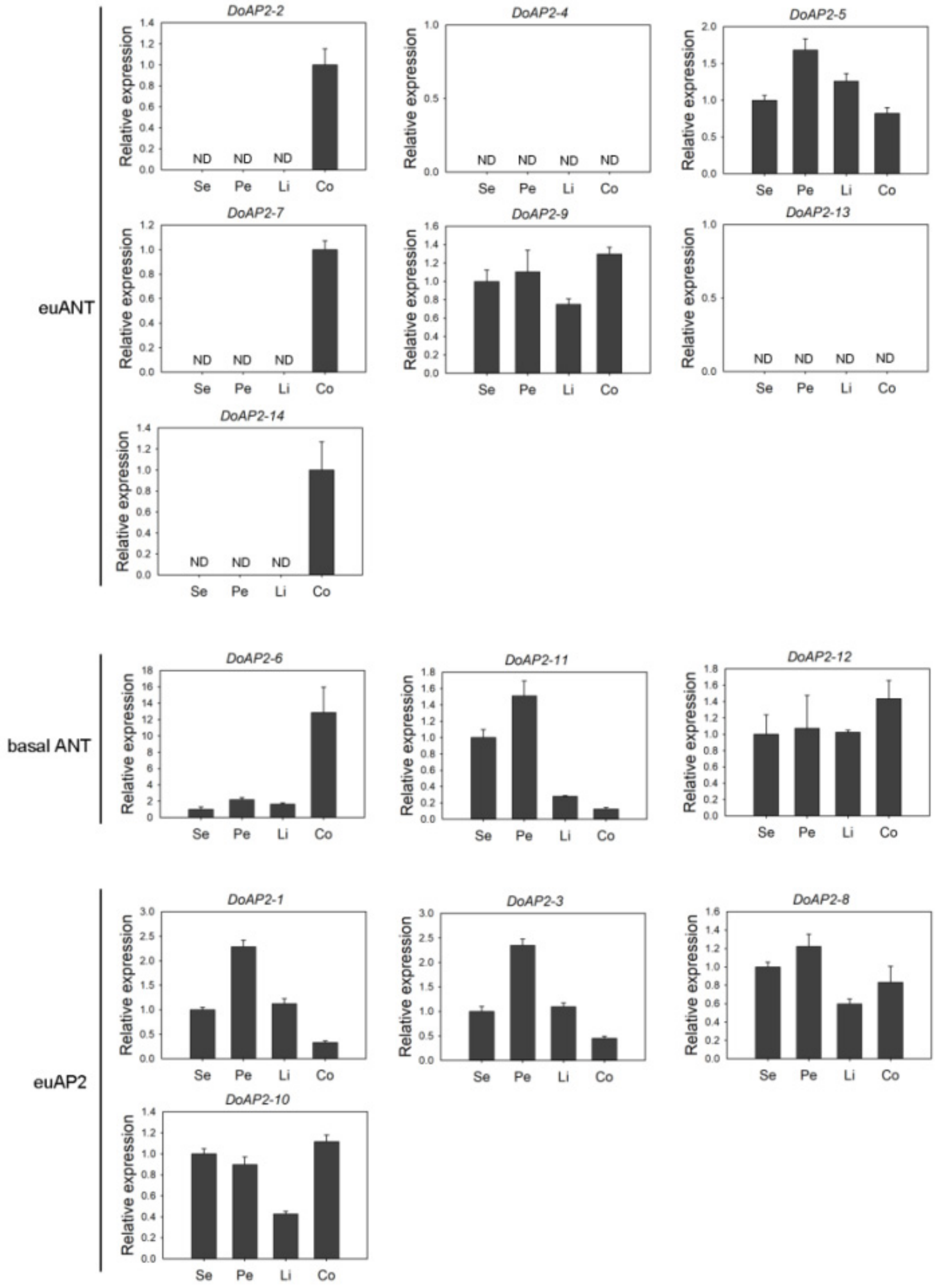

Figure S2. Expression analysis of *DoAP2* genes in different tissues of FBF by qRT-PCR. FBF, fully bloomed flower; Se, sepal; Pe, petal; Li, lip; Co, column. Each data bar represents the mean ± standard deviation (SD) of three biological replicates (*n*=3). ND, not detected.

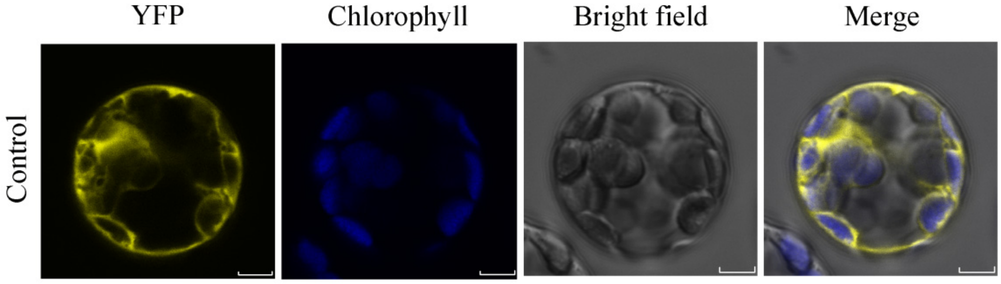

Figure S3. Subcellular localization of positive control (empty YFP vector) in *A. thaliana* protoplasts. Bars = 5 μm.

Table S2. Primers used for qRT-PCR.

Primer name	Primer sequences (5′→3′)
DoAP2-1F	GGATGAAATCCGTGTTGCTT
DoAP2-1R	AACGTGACTCCCCGATACTG
DoAP2-2F	AGCAGGTAAGGGAGTGCAGA
DoAP2-2R	CGCTCTTGCAGCTTTCTCTT
DoAP2-3F	ACACCAGGTTCGGATTGAAG
DoAP2-3R	CAGCATGAGCTGTGTCGAAT
DoAP2-4F	GGGCCACCTACAACGACTAA
DoAP2-4R	CGCATCTTCCTGTGTGCTAA
DoAP2-5F	AACCTATCCGCCACAGAGTG
DoAP2-5R	CATCCTAACGAACCCTCCAA
DoAP2-6F	AGGCCAATCCAAGGAAGAGT
DoAP2-6R	GACCGCGATATTCAATTGCT
DoAP2-7F	CCCACTACCACGACCAATTT
DoAP2-7R	CTTGTTGCCGGCTACTCTTC
DoAP2-8F	TCCGGTTTGGTATCCTCAAG
DoAP2-8R	CGCCTGCTTTTCTTTACCTG
DoAP2-9F	TTCTATGCCTTCCGGATTTG
DoAP2-9R	GTGCTTCGAATTTCCCACTC
DoAP2-10F	TAGAATGGGCCAGTTCCTTG
DoAP2-10R	TGAGCTCCCCAGACTCAGAT
DoAP2-11F	GACATGGCCGCCTTAGAATA
DoAP2-11R	CGCAATCAAACAAATCATGG
DoAP2-12F	AATACTGGGGCCAAGAAACC
DoAP2-12R	ATATGCAGTTGCAGCCTCCT
DoAP2-13F	TCAGGAGGAAGAGCAGTGGT
DoAP2-13R	AAGGGTGCTGCTCTCCAGTA
DoAP2-14F	CCAATTCCAATCGTTTGACC
DoAP2-14R	GTTGGCCATCCTCGAAGTTA

Table S3. Primers used for subcellular localization analysis.

Primer name	Primer sequences (5′→3′)
YFP-DoAP2-1F	AGCTCAAGCTTCGAATTCATGGTGCTAGATCTCAACGTG
YFP-DoAP2-1R	CCGTCGACTGCAGAATTCGCCCTGGAAGAAGCCATGGC
YFP-DoAP2-2F	AGCTCAAGCTTCGAATTCATGAAACCCATGAGCGGTAG
YFP-DoAP2-2R	CCGTCGACTGCAGAATTCAGCATCACTCCATCCTGAATAC
YFP-DoAP2-6F	AGCTCAAGCTTCGAATTCATGGCGAAGAAGAAGCCGAAC
YFP-DoAP2-6R	CCGTCGACTGCAGAATTCTAAATCAAGCTCACATGGG
YFP-DoAP2-11F	AGCTCAAGCTTCGAATTCATGGAGAAATCAAATTCTTC
YFP-DoAP2-11R	CCGTCGACTGCAGAATTCAGAGTATATACTGACTGGAGG

Table S4. Primers used for the dual-luciferase reporter gene system.

Primer name	Primer sequences (5′→3′)
pBD-DoAP2-1F	TCGCCGACCGGTAGGCCTATGGTGCTAGATCTCAACGTG
pBD-DoAP2-1R	AACCAGAGTTAAAGGCCTGCCCTGGAAGAAGCCATGGC
pBD-DoAP2-2F	TCGCCGACCGGTAGGCCTATGAAACCCATGAGCGGTAG
pBD-DoAP2-2R	AACCAGAGTTAAAGGCCTAGCATCACTCCATCCTGAATAC
pBD-DoAP2-6F	TCGCCGACCGGTAGGCCTATGGCGAAGAAGAAGCCGAAC
pBD-DoAP2-6R	AACCAGAGTTAAAGGCCTTAAATCAAGCTCACATGGG
pBD-DoAP2-11F	TCGCCGACCGGTAGGCCTATGGAGAAATCAAATTCTTC
pBD-DoAP2-11R	AACCAGAGTTAAAGGCCTAGAGTATATACTGACTGGAGG
